# Novel use of social media to assess and improve coastal flood forecasts and hazard alerts

**DOI:** 10.1038/s41598-021-93077-z

**Published:** 2021-07-02

**Authors:** J. M. Brown, M. J. Yelland, T. Pullen, E. Silva, A. Martin, I. Gold, L. Whittle, P. Wisse

**Affiliations:** 1 National Oceanography Centre, 6 Brownlow Street, Liverpool, L3 5AD UK; 2 National Oceanography Centre, European Way, Southampton, SO14 3ZH UK; 3 HR Wallingford, Howbery Park, Wallingford, Oxfordshire OX10 8BA UK; 4 Sefton Council, Trinity Road, Bootle, Liverpool, L20 3NJ UK; 5 Environment Agency, Richard Fairclough House, Knutsford Road, Warrington, WA4 1HT UK

**Keywords:** Environmental impact, Physical oceanography

## Abstract

Coastal communities and infrastructure need protection from flooding and wave overtopping events. Assessment of hazard prediction methods, used in sea defence design, defence performance inspections and forecasting services, requires observations at the land-sea interface but these are rarely collected. Here we show how a database of hindcast overtopping events, and the conditions that cause them, can be built using qualitative overtopping information obtained from social media. We develop a database for a case study site at Crosby in the Northwest of England, use it to test the standard methods applied in operational flood forecasting services and new defence design, and suggest improvements to these methods. This novel approach will become increasingly important to deliver long-term, cost-effective coastal management solutions as sea-levels rise and coastal populations grow. At sites with limited, or no, monitoring or forecasting services, this approach, especially if combined with citizen science initiatives, could underpin the development of simplified early warning systems.

## Introduction

Industry standard methods of estimating wave overtopping at a sea defence^1, 2^ involves transforming the offshore wave data (from buoys or numerical models) to the structure’s toe. EurOtop^3^ is the industry manual (freely available at www.overtopping-manual.com), which provides a standard description to assess the mean overtopping (q) for a range of structures using empirical rules derived from test data. Bayonet GPE^4^ is a free to use online tool (www.overtopping.co.uk) that accompanies EurOtop to predict the overtopping discharge (l/s/m) for a given structure and the wave and water levels at the structure toe. It applies a statistical technique (Gaussian Process Emulator—GPE) to the EurOtop empirical data to predict both the overtopping discharge and the uncertainty in that prediction. Globally adopted hazard thresholds for pedestrian and vehicle safety^3^ are then used to set design requirements and operational alert thresholds for coastal management schemes and hazard forecasting services^5^.

The overtopping database^6, 7^ underpinning EurOtop and Bayonet GPE includes particular defence structures and environmental conditions. The data are largely based on experiments performed under idealised flume conditions^8^, supplemented by extremely limited field data^9^ (mostly obtained at dykes^10^). Sea defences are designed using overtopping simulations for a limited range of water level and offshore wave conditions. Flood forecast systems are also often designed using look-up tables for a particular range of conditions, especially when computational resources are limited. In both cases, the conditions chosen usually represent the more extreme, low-probability events that may occur during a structure's approximate 100 year lifetime. This means that more typical, frequently-occurring conditions are not considered even though they may pose a hazard to pedestrians, vehicles, property or infrastructure. If the specific structure geometry and/or combination of hydraulic conditions are not included in the overtopping database used to train Bayonet GPE, then overtopping predictions can have orders of magnitude of uncertainty.

In addition to the limitations of the overtopping database, there are two important environmental factors that are often lacking in standard overtopping tools. Firstly, on-shore winds can increase the amount of water driven over the defence: neglecting this effect may lead to an under prediction of the overtopping^11^. Secondly, the beach level near the toe of the structure evolves, with large changes sometimes occurring rapidly after a severe storm. Such changes can noticeably alter the overtopping for a given combination of water level and wave conditions^12^, but industry standard methods are usually based on a single beach level.

When designing a look-up table based forecast service, or designing a coastal structure, it is not practical (computationally feasible) to expand the range of simulations to include all possible water level, wave, beach level, and wind conditions. Alternative methods of assessing the effectiveness of a defence, and the accuracy of flood and hazard forecasting systems, are therefore required.

Coastal monitoring techniques have evolved greatly in the latter half of this century, particularly with remote sensing techniques and more recently crowd-sourced data^13^. With the growth of citizen science programmes in recent years, new challenges to integrate the observations into coastal management activities and coastal conservation are emerging^14, 15^.

This paper demonstrates how social media content can be used to build a database of the different conditions (water level, offshore waves, wind) that cause overtopping at a specific site. This allows us to:

1. Identify the primary environmental conditions that drive overtopping;
2. Quantify the range of conditions that should be considered in flood hazard prediction tools used in forecasting services, hazard response plans and structure design;
3. Demonstrate how local hazard response activities can be more effectively focussed.

This approach could be applied to any site where there is a sufficient footfall. In future, citizen science initiatives could be set up where people are encouraged to (safely) take photographs of overtopping from a fixed location and upload them to a dedicated website.

## Results

**Figure 1:**
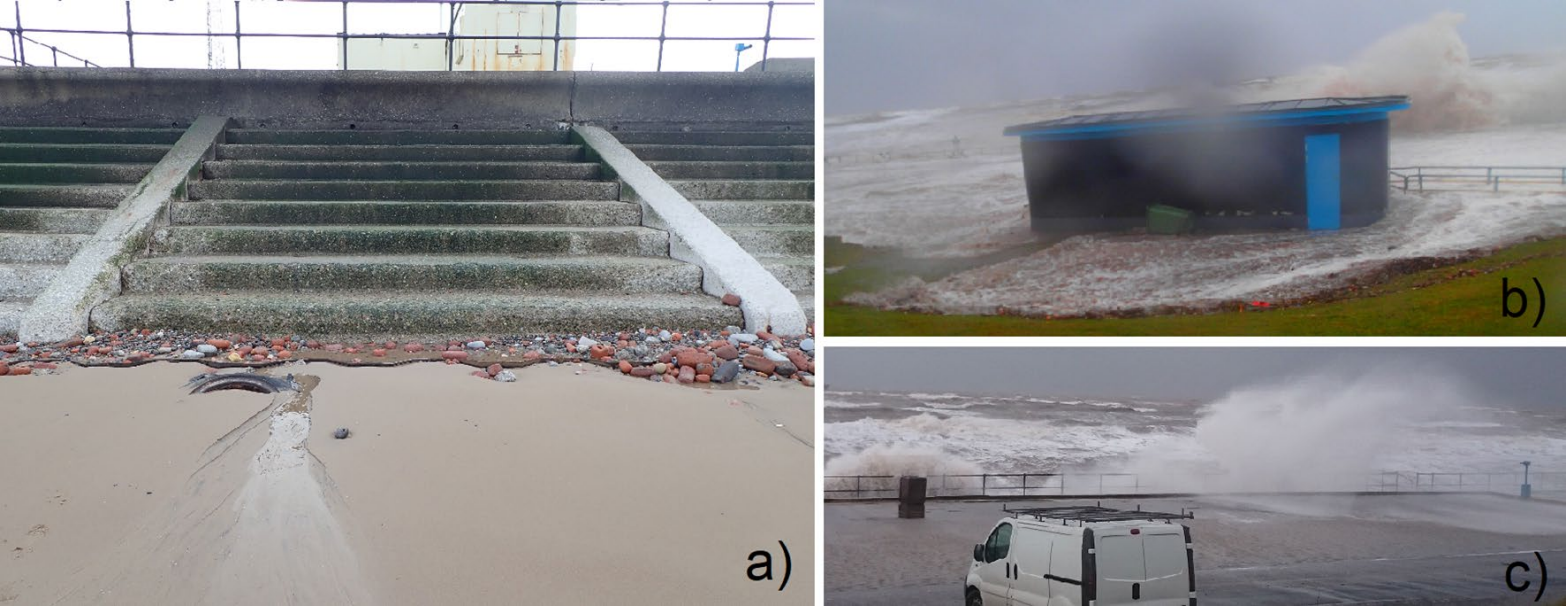
The case study site. (**a**) North Crosby beach, where the ageing coastal defence comprises a stepped revetment fronting a recurved sea wall. The promenade with a splash wall fronting a public car park is sometimes flooded, e.g. on (**b**) 5th December 2013, or partially flooded with the overtopping waves posing a hazard to people on the promenade and the seaward parking spaces, e.g. on (**c**) 8th February 2016.

**Figure 2:**
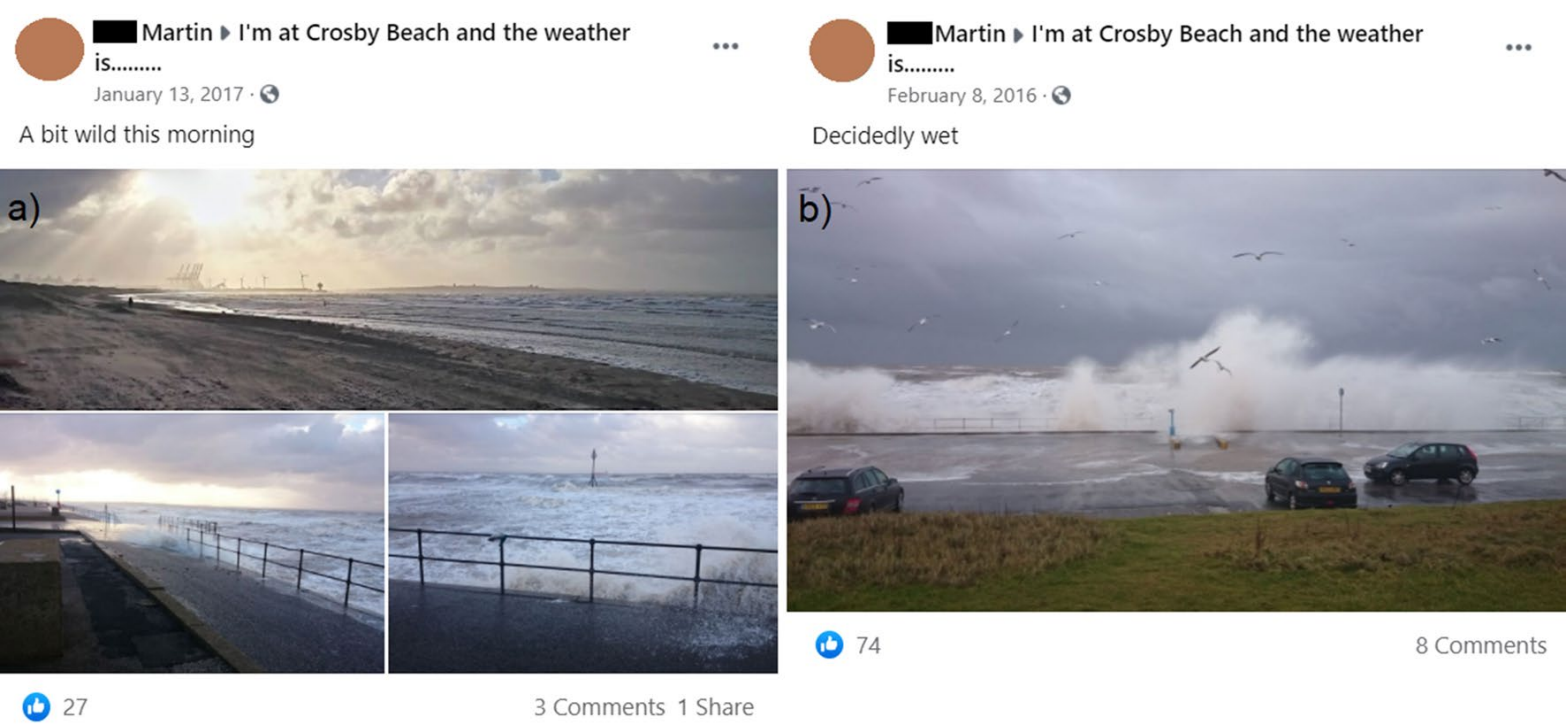
Facebook posts by author A. Martin showing examples of different severity wave overtopping at Crosby between 2013 and 2017. (**a**) Low magnitude overtopping over the promenade and (**b**) extreme overtopping posing a hazard to the car park.

Our case study site is Crosby, Northwest England, where a 900 m long sea defence delivers the “hold the line” shoreline management policy to protect a coastal town and associated infrastructure. The site is exposed to a tidal range of 10 m (i.e. is hyper-tidal) and significant wave heights up to 5.5 m^16^. The beach is wide with a low gradient and the overtopping hazard is made worse due to the debris carried by the waves. The northern section of the sea defence includes a stepped revetment, vertical wall with a recurve forming the seaward edge of a 4 m wide promenade at a height of approximately 6.4 m AOD and a rear splash wall at a height of 7.2 m AOD (Figs. 1a and 2a). Flood hazard management at Crosby, and most other locations, relies on the managing authorities having knowledge of the conditions that cause flooding, or a hazard to people and vehicles on the seafront, or damage to the defence itself, and how these conditions may change in the future.

Like many popular coastal destinations, Crosby has dedicated social media channels. For example, an open access community Facebook page^17^ (“I’m at Crosby beach and the weather is….”) was set up in 2013 during an exceptionally stormy winter^18, 19^ (Fig. 1b). We used photographs from the page to identify the dates when wave overtopping of any magnitude occurred over the 2013–2017 period (Fig. 2). The water level, wind and wave conditions for these periods were extracted from national monitoring networks and used as inputs to Bayonet GPE to predict the mean overtopping discharges for the observed events. Some of the events resulted in a prediction of zero overtopping: this may be due to gaps in the overtopping database for low severity events, and/or to neglecting the effects of on-shore winds.

### The use of joint probability curves

**Figure 3:**
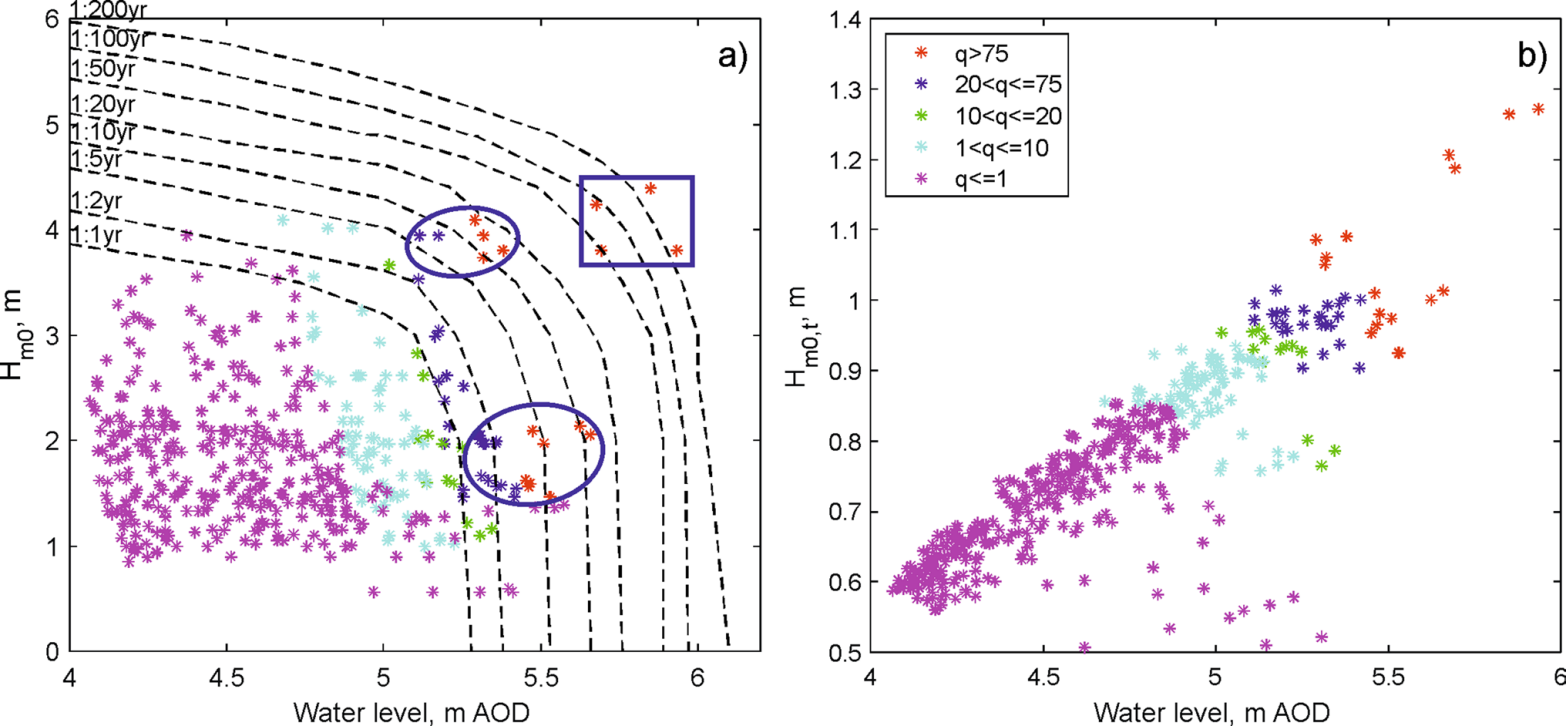
(**a**) The joint probability curves^20^ used to identify the storm event severity at Crosby. The blue square indicates the extreme conditions that are likely to be considered for future sea wall design. Also shown are our mean overtopping discharges (q, l/s/m) for the Facebook events. The blue circles indicate overtopping events that occur relatively often (1 in 2 year) and are hazardous to pedestrians and vehicles, but are missed from the standard approach that focusses on low-probability extreme events. (**b**) Wave heights at the toe of the structure have a direct influence on overtopping (colours) and are limited by the water level (Above Ordnance Datum, AOD).

Joint probability analysis of total (tide plus surge) water levels and offshore (zero momentum) wave heights (H_m0_) is often used to classify event severity and assess a structure’s long-term performance against its design specifications (Fig. 3a). Sea wall design focuses on extreme, low probability conditions (often 1 in 200 year). This focus on extreme conditions misses large overtopping events when the offshore waves are small but the water levels are high: conditions with a 1 in 5 year joint probability of occurrence (Fig. 3a) that result in hazardous overtopping of similar magnitude (q of more than 75 l/s/m) to that of more extreme, low-probability events. Even annual events can exceed the 10 to 20 l/s/m thresholds^3^ for the safety of pedestrians. Another issue with this approach is that at Crosby the waves are depth-limited. This means that the wave heights at the structure toe (H_m0,t_) have most effect on the overtopping (Fig. 3b), not the offshore wave conditions (Fig. 3a). Water level is thus a key factor in controlling the overtopping hazard at Crosby.

### The environment agency flood warning system

The models and tools used for new scheme design^1^ also form the basis of regional flood forecasting methods^2^ used as part of the Environment Agency’s (EA’s) national flood forecast and hazard alert service. The EA share a 5-day forecast to first responders^21^, with alerts and warnings issued less than 12 h ahead, at vulnerable locations around the English coast: one of the locations is positioned in the Crosby car park (Fig. 1). Alerts are issued for relatively minor (less than 1 in 20 year) flooding events that, at Crosby, may impact the seafront and car parks. Warnings are issued for more severe events that may pose a hazard to property: at Crosby the houses are set well back from the seafront so these events are rare. The system uses forecast data for wind, wave and water level conditions along with the industry-standard numerical tools^7^ for predicting overtopping. The wind conditions are used in the local wave transformation models only: they are not used in the overtopping tools. On 5th December 2013 (Fig. 1b) Crosby experienced one of the most extreme flood events on record^19^, but no flood alerts or warnings were issued. As a result, the EA refined the local safety thresholds: since 2013/2014 an overtopping alert or warning is issued for Crosby if the mean overtopping discharges exceed 2 l/s/m or 25 l/s/m respectively. Some of the later Facebook events clearly showed overtopping that posed a hazard to pedestrians (e.g. Fig. 1c) but the EA rarely, if ever, issued alerts at Crosby in the 2013–2017 period examined.

**Figure 4:**
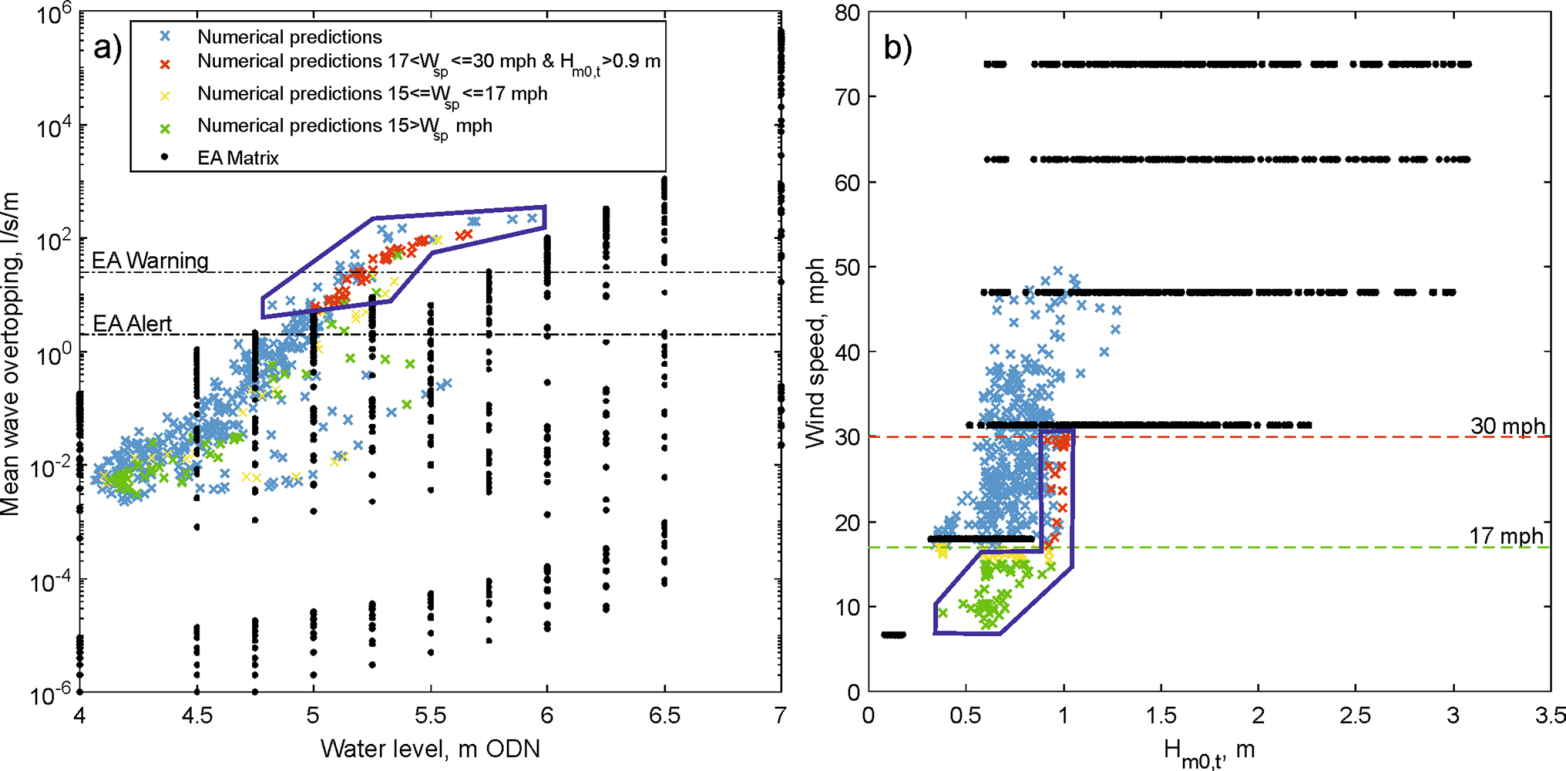
The EA matrix (black) and our overtopping predictions for different wind speeds (W_sp_) as shown in the key. (**a**) Overtopping discharges for different water levels. The blue polygon indicates the hazardous Facebook events that are under predicted in the EA matrix. (**b**) The range of wave and wind conditions considered. The blue polygon highlights the wave conditions (green, yellow and red crosses) which can produce hazardous overtopping when water levels exceed 5 m AOD, but are out of range in the matrix.

Figure 4 shows all the water level, wind speed and wave conditions that are included in the EA forecast matrix along with the EA predicted overtopping. We used the same industry-standard methods to simulate the overtopping for the conditions that occurred during the Facebook events for comparison. However, the EA predictions were based on a 2009 beach profile, whereas we used a profile obtained in February 2017. Figure 4b shows that there is a lack of moderate wind (less than 17 mph) and moderate wave (0.5 m to 1 m at the toe) combinations within the EA’s matrix. These breezy spring tide conditions are often associated with "nuisance" overtopping, but it can be seen that on some occasions an alert or warning should have been issued according to our predictions. More importantly, the overtopping predictions in the EA matrix are biased low relative to our predictions for the observed events (Fig. 4a).

**Figure 5:**
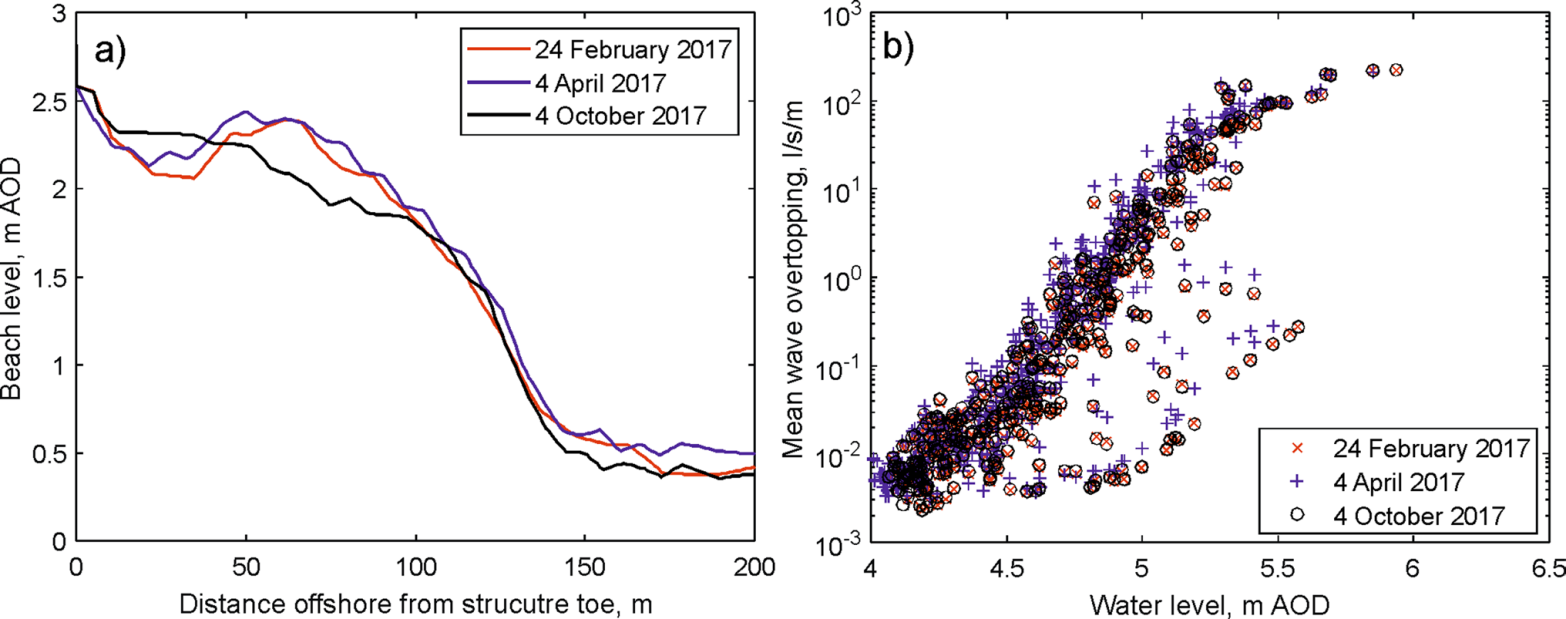
(**a**) Beach profiles collected during 2017. (**b**) Overtopping predictions calculated for each profile, showing consistently larger overtopping in April compared to February and October (note the logarithmic y-axis scale).

Figure 5a shows three different beach profiles obtained during 2017. The April profile shows that the beach level within 10 m of the structure was about 0.15 m lower than in February and October of that year: this small change resulted in consistently greater overtopping predictions for a given water level (Fig. 5b). In contrast, changes in the beach profile more than 10 m from the structure have minimal impact. The 2009 beach level used in the EA’s forecast system is approximately 1.5 m higher at the toe than the 2017 beach profiles. This large change in beach level would result in significantly smaller overtopping predictions (for a given wave and water level combination) than those shown here using the 2017 profiles, and would explain the lack of alerts from the EA. In contrast, a profile obtained in 1996 showed that the beach level near the structure was up to 0.45 m lower than in 2017 and would have resulted in much greater overtopping predictions.

### The local authority hazard monitoring and response system

Due to a lack of EA alerts at Crosby, the Local Authority (LA) have developed an early warning system for hazard monitoring, performance inspections and response. For example, planning: closures of public car parks if there is a risk of flooding; defence inspections after hazardous events to assess the flood resistance and fragility of the ageing sea wall; beach surveys after extreme events to assess if beach lowering has made the structure toe vulnerable. Maintenance inspections are performed annually and also after storm events.

Previous research^22^ using model simulations for the natural (sand dune) coast north of Crosby provided key information about hazardous water level and wave conditions. These were adapted for the structure at Crosby and are currently applied as hazard warning criteria when the wind is between Southwest and Northwest as follows:

- WL + ½ H_m0_ ≤ 7.2 m AOD, no hazard
- WL + ½ H_m0_ > 7.2 m AOD, hazard to promenade users and car park
- WL + ½ H_m0_ > 7.6 m AOD, likely car park closure due to flooding

where WL = total (tide plus surge) Water Level (m AOD, Above Ordnance Datum) and H_m0_ is the offshore zero moment wave height (m). The thresholds are based on the splash wall level (7.2 m AOD, Fig. 2a) fronting the car park and the toilet block platform level (7.6 m AOD, Fig. 1b) situated at the inland edge of the car park.

**Figure 6:**
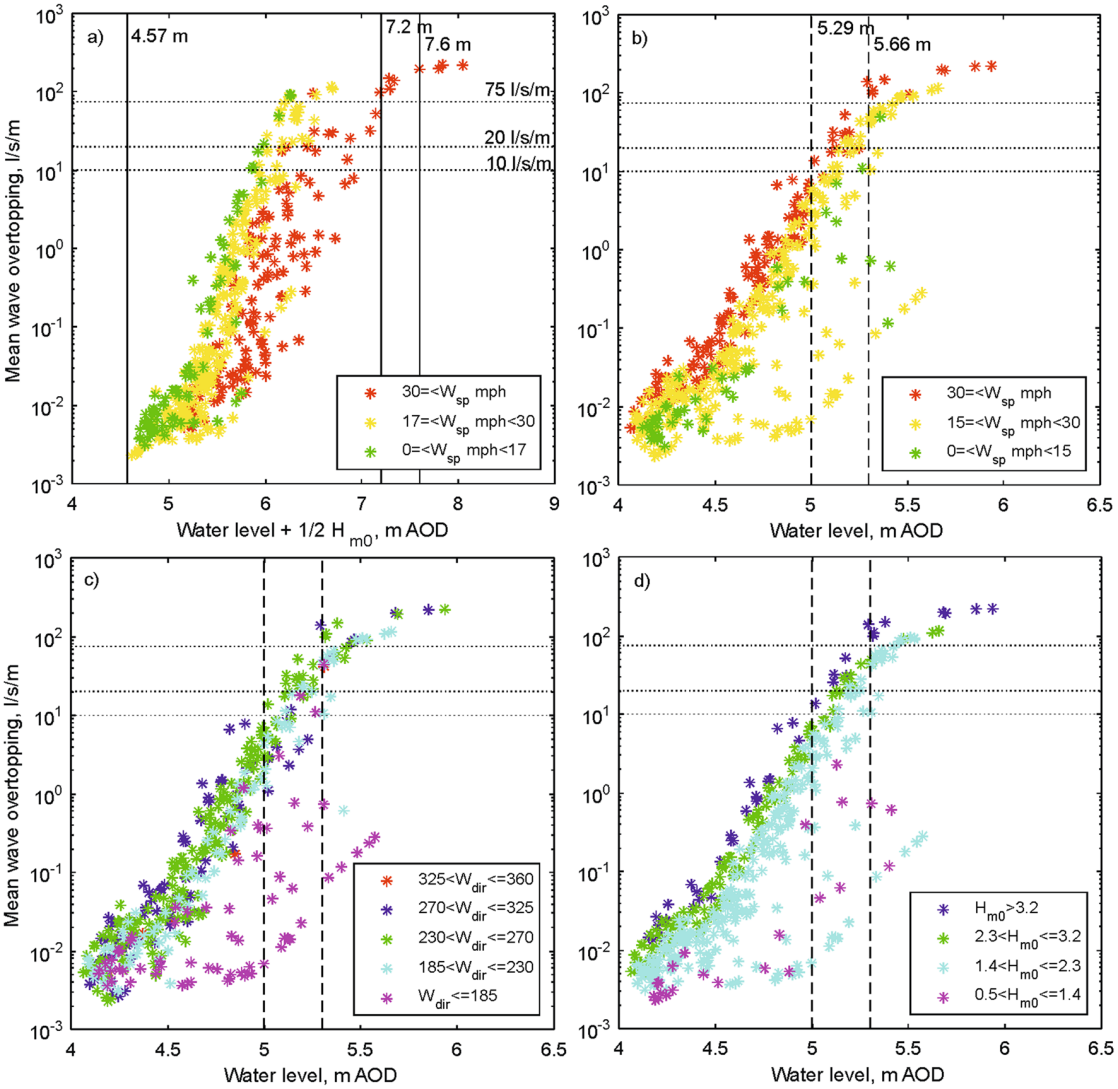
Predictions of overtopping for the Facebook events. The horizontal lines show the EurOtop hazard thresholds for pedestrians (10 to 20 l/s/m) and vehicles (20 to 75 l/s/m). (**a**) The typical management approach to hazard classification (vertical lines indicate the hazard criteria), based on a combination of water level and offshore wave heights. The colours show wind speed classes for onshore wind directions. In contrast, the operational approach of the LA is based on water level without offshore wave heights. The colours show the influence of (**b**) wind speed (W_sp_) and (**c**) wind direction (W_dir_), and (**d**) the associated offshore wave heights (H_m0_).

To assess this approach we again use our predictions of overtopping for the 2013–2017 Facebook events. Figure 6a shows the predicted overtopping for the different hazard criteria that are based on combining water levels and offshore wave heights. For a given set of wave and water level conditions, lower wind speeds appear to cause more overtopping than higher wind speeds: this unphysical, misleading artefact is caused by the waves at the structure toe being depth-limited at this site. On windy days the offshore wave heights are larger (so those data are moved to the right on the x axis in Fig. 6a), but these waves break as they propagate into shallow water, i.e. become depth-limited (Fig. 3b), and do not necessarily result in significantly greater overtopping than is seen on less windy days with the same water level (Fig. 6b). For this reason the 7.2 m AOD threshold for the combined wave and water level height is very rarely reached, even though conditions which cause a hazard to pedestrians or vehicles occur quite often.

In practice, the LA's operational hazard criteria omit offshore waves and use only easily-accessible tidal predictions and forecasts of surge and wind conditions to trigger a "traffic light" system of hazard alerts or warnings (Fig. 6b):

- No hazard: 4.57 m AOD < WL < 7.2 m AOD, wind speed ≤ 16 mph from west to north west
- Alert: 4.57 m AOD < WL < 7.2 m AOD, 16 < wind speed < 30 mph from west to north west
- Warning: 4.57 m AOD < WL < 7.2 m AOD, wind speed ≥ 30 mph from west to north west
- Warning: WL ≥ 7.2 m AOD, any wind condition

where 4.57 m was identified^22^ as a critical level and is similar to mean high water spring tide (4.46 m AOD).

By considering the mean overtopping discharge against water level alone (Fig. 6b), it is more clearly seen that in this depth-limited location higher wind speeds (with a westerly component, Fig. 6b,c) cause more overtopping. It also confirms that neglecting offshore wave conditions (Fig. 6d) is not detrimental, since the small influence of offshore wave heights is well represented by the inclusion of wind speed: this would be expected since the offshore wave heights are driven by westerly to north-westerly winds. However, we suggest that the traffic light system could be simplified by using a wind speed of 15 mph for all thresholds. It could also be improved by changing the water level thresholds to better represent the degree of hazard, i.e. a water level threshold of 5.29 m for hazards to pedestrians rising to 5.66 m for hazards to vehicles in the car park (Fig. 6b). It should be noted that these water level thresholds will vary with the beach profile (see above) and should be reviewed if the beach level changes significantly.

## Discussion

Overtopping rates are governed by the water levels for this depth-limited hyper-tidal location and the wave heights at the structure toe, rather than offshore, mediate the overtopping discharge. This means that the use of joint probability analysis based on offshore wave heights may have limited applicability in such depth-limited locations. For Crosby we show typical (high probability) events can generate the same overtopping hazard as extreme (low probability) events, and should therefore be considered in hazard response plans (both societal and structural). This also has implications for engineering works, as the wave overtopping discharges of the magnitude associated with more extreme coastal conditions used in the structure design may occur much more frequently than expected. This could potentially result in unexpected under-performance when assessed against the design criteria, or even structural damage.

Using the database of Facebook events for Crosby, we assessed the LA's hazard alert process and suggest simplifications and improvements that would make it more effective. We found hazardous overtopping occurred much more frequently than expected. Similarly, we showed that at this site the EA flood forecasting system fails to predict the vast majority of hazardous overtopping events since:

1. The height of the beach within about 10 m of the toe of the defence has a very strong influence on the overtopping for any given wave and water level conditions. Use of a high beach level results in very large under-predictions of overtopping when the beach level is in reality tens of centimetres lower (typical of seasonal variability). Conversely, use of a profile that is too low would result in numerous false alerts.
2. Moderate wind conditions frequently cause hazardous overtopping, but such conditions are currently poorly represented in the EA matrix.

These results showed that the EA system, based on industry standard approaches to flood hazard prediction, could be significantly improved by being:

1. Updated to include overtopping predictions based on a range of beach profiles (at least an upper and lower) to account for variability in the beach level, both over time and along the length of the defence. The forecast system should have a design life, with performance review intervals, particularly as sea levels rise further.
2. Expanded to include more typical conditions that are shown here to cause overtopping but are currently missing.

As a result of this work, the EA plan to include these modifications in the next upgrade of their national flood forecast system.

## Conclusions

We have shown that information harvested from growing social media records can be used to build a database of conditions (obtained from national wave, wind and water level monitoring networks) that cause overtopping at a particular site. When combined with industry standard tools for estimating total overtopping discharges the database can be used to quantitatively assess and improve local flood forecasting and hazard management strategies. This approach also provides a database of overtopping information, which is currently lacking since forecast services do not usually archive overtopping predictions.

Our social media approach could be applied to any popular coastal site, or even to inland locations with sufficient footfall, to support hazard manamgemt decisions. At sites where there are no hazard forecasting systems in place, the approach could be used to establish the critical environmental conditions that cause overtopping, and monitor how these change in the long term. The basic approach discussed in this paper could be developed further by:

1. Classifying the overtopping images into those that show (qualitatively) hazardous events and those that show only nuisance/non-hazardous events, and hence set suitable hazard thresholds and issue alerts or warnings using publicly available wind, water level and wave forecasts. This simplified approach would remove the need to run models and access industry tools in order to quantify overtopping discharges for hazard monitoring and operational response.
2. Instigating a more systematic citizen science approach. For example, the existing international CoastSnap programme^23^ monitors coastal erosion using fixed posts with mounts for smart phones (https://www.coastsnap.com/). Signs encourage the public to take photographs of the beach and upload them to a dedicated website. The images are analysed to provide time series of changes to the coastal morphology. A citizen science program focussed on (safely) obtaining overtopping images could be developed independently of, or in partnership with, CoastSnap.

In this way, citizen science or social media images could be used to develop a growing evidence base to assess the changing performance of sea defences and hazard forecasting systems over time. It could also raise public awareness of changing coastal hazards and build a community consensus when planning coastal climate resilience strategies^24^. Finally, the images could also provide a near real-time (within roughly 24 h) data source to visually validate, re-calibrate and improve hazard warning systems.

## Methods

We use Crosby (Northwest of England) as a case study site. Here, like other locations, the Victorian sea wall is nearing the end of its design life while wave overtopping is increasing with the rising trend in high water level observed at Liverpool^25^. Crosby provides a good illustration of the many coastal sites where increased understanding of the present-day hazards and refinement of flood forecast and hazard alert services would be of value.

Like many popular coastal locations, Crosby has dedicated social media channels including a community Facebook page^17^ (“I’m at Crosby beach and the weather is….”, https://en-gb.facebook.com/groups/526198870745222/) that contains numerous photographs that often capture overtopping at the sea wall. At this site high water spring tides occur close to mid-day when the visitor numbers are often high and are increased by people in the local area taking a lunch break with a view. When large waves are overtopping the promenade people typically watch the sea from their cars or stand in the car park at a safe distance from the overtopping discharges. After joining the Facebook group, each photograph posted from December 2013 to December 2017 (inclusive) was visually inspected. Most of the photos were taken from, or close to, the Crosby (Hall Road) beach car park, one of the main access points. Any photos with some level of overtopping at the Crosby sea wall had their comments checked to verify the date of the photo. In this way a set of dates when overtopping was observed was collated. While media reports can potentially be used to classify storm severity for some events^26^, we do not attempt to classify the overtopping in the images, but simply use them as a yes/no for the occurrence of overtopping.

The social media record is thus used to identify overtopping events in the four year period since December 2013. It captures the winter 2013/2014, when the worst recorded storm events at Crosby occurred: the event on the 5th December 2013 exceeded the 1 in 200 joint probability of annual occurrence^19^. Our study thus covers a range (see Fig. 2) of conditions from bright and breezy days to the most extreme events when coastal managers (who also post on local community media channels) are on site in response to hazard warnings. The public appeal of waves and overtopping means that social data sources often provide good coverage of typical to extreme conditions. The Facebook events were complemented by additional photos taken by coastal managers and practitioners during site inspections.

For each date associated to a Facebook image, the environmental conditions that prevailed at that time were obtained to form our database of forcing conditions. The database was based on national monitoring systems, and includes: 30 min offshore wave data from the Liverpool Bay wave buoy (24 m depth); 15 min water levels from the Liverpool Gladstone Dock tide gauge; bi-annual and post-storm beach surveys (at Hall Road car park) that are extended offshore by the most recent (2010) bathymetric data; and, hourly wind data from the weather station at the coastguard station at the car park entrance. One of the longest beach transects (ref no. 11A02250) regularly surveyed by the LA extends about 900 m offshore from the car park entrance. This allows a computationally efficient cross-shore wave model transect to be set up, extending from the structure toe to a depth of 24 m (19 km offshore). The model transect is orientated in a westerly direction towards the open sea, and best aligns with the dominant westerly to north westerly wave directions, and can be forced directly by available monitoring. The majority of the shore-normal transects fronting the structure are oriented more southerly towards the adjacent Wirral Peninsula. The offshore wave data are available less than 10 km north of our transect. The environmental data are extracted for a 12.5 h (tidal) window centred at the time of high water on the day when the photo was taken. Conditions are also collected for the tides prior to and after our window, which occur outside daylight hours. Hence, if a storm lasts for multiple tides we capture this information. The data were linearly interpolated to 15 min intervals consistent with the tide gauge record. Only periods when the water level exceeded the elevation of the structure toe (2.58 m AOD, the lowest beach level from all surveys between 1996 and 2017) were considered for further analysis. This created a dataset of 1244 conditions that covered periods when some level of overtopping was likely to have occurred.

The 15 min environmental (wave, water level and wind) data provide the input for a shallow water wave model (SWAN^27^), used to transform the offshore wave conditions to the toe of the sea wall structure. SWAN, run in 3rd generation and stationary modes, was calibrated/validated using coastal waves and water levels measured by an Acoustic Waves And Current (AWAC) instrument deployed in 2017 at the low water mark seaward of the Hall Road car park. The intertidal profiles used to form the model transect, for the results presented here, were from 2017 (24th February, 4th April and 4th October). The identified overtopping events in 2017 closest to the survey dates used to calibrate SWAN occurred 25th February, 4th April and 4th October. The first event was used to calibrate SWAN and the latter events to validate the parameter settings. Parameters in the model were set using: available observations, a previous storm modelling study in Liverpool Bay^16^, or default settings where there was no information. The best results (within 10% of the observations) were obtained for a bottom friction setting that used bed ripples based on a sediment size of 0.23 mm, the median grain size for the upper beach^28^. Given no currents were considered and a transect approach rather than 2-dimentional horizontal grid was applied this was acceptable. The horizontal grid resolution was 10 m to allow an efficient run time and provide the required wave height (H_m0_), wave period (T_m-1,0_) and water depth conditions within 5 m of the structure toe for input into Bayonet GPE for the numerical assessment of the mean wave overtopping discharge (l/s/m).

The assumptions made to set up Bayonet GPE^29^ were based on the structure profile obtained from a laser scan in 2013. The empirical rules in EurOtop for a smooth dyke slope, wall and bullnose structure were applied to represent the Crosby structure. A variable friction to account for the steps on the sloped revetment was set depending on the water level. An influence factor for the permeability and roughness of, or on, the slope of 1 represents smooth concrete and 0.75 represents the influence of ribs. As the steps have a greater relative roughness at low water levels (i.e. the wave has to travel over more steps so the friction is greater) and at high water the steps are less effective at retarding the run up, we apply a roughness factor of 0.9 when the water reaches the top step and 0.75 when the water levels are near the toe, linearly interpolating between these values for intermediate water levels. Wave angle was not considered for the short-crested conditions at Crosby, where the wave approach is from an acute angle. Along with the mean discharge, Bayonet GPE calculates the upper and lower 1st and 2nd standard deviations about the mean value along with a confidence value (a Mahalanobis distance "md"—a measure of closeness to the training data). An md value of less than 2 indicates high confidence; data with md greater than 2 are discarded and only data with md less than 2 are shown in this paper. It should be noted that these overtopping predictions could be biased low due to the omission of wind influence: in particular, on-shore winds can mitigate against the effect of the sea wall's return curve and drive a vertical wave plume over the structure crest.

A range of known wave and water level conditions were simulated in the laboratory: a physical model of the Crosby sea wall and beach was created within one of HR Wallingford’s wave flumes at a scale of 1:7.5. Mean overtopping discharges, q, were measured for experiments lasting over 1000 waves for the various wave and water levels. The results were found to be in good agreement with the predictions obtained using Bayonet GPE^30^ and thus gives confidence in our numerical approach.

The numerical Facebook results^31^ presented in this paper for the period 2013–2017 focus mainly on the overtopping predictions using the winter profile collected on 24th February 2017 (although some results are shown for the other two profiles obtained in 2017). The numerical SWAN-Bayonet GPE approachl produced up to 16 overtopping predictions per high tide window, covering about 2 h either side of high tide when the water levels enabled wave overtopping to occur. In total, 465 environmental condition combinations confidently predicted overtopping of some level: these form the Facebook data plotted within this paper that are available through the British Oceanographic Data Centre^31^.

Our approach follows a similar SWAN-EurOtop approach as used by the EA in 2009 to generate the lookup tables (assessed here) in the flood forecasting service for the North West region, namely TRITON. The numerical approach is both computationally efficient and utilizes available coastal monitoring. TRITON^21^ has long been considered the best practise for flood forecasting in England and Wales and is used operationally. TRITON provides flood forecasts for the Crosby (Hall Road) beach car park and the only adjustment made was the alteration of the hazard threshold levels following the extreme events in 2013, which did not trigger a warning. The 2009 beach-structure profile, used to create the operational lookup tables for this location, is located approximately 125 m south of the transect applied in our numerical approach. The numerical approach applied in this study is therefore appropriate to assess the predicted overtopping discharges based on lookup tables for a range of plausible nearshore conditions at this location. This numerical approach will also be adopted by the consultant assessing the business case for a new scheme at Crosby, when considering statistical extremes in nearshore conditions and future sea levels. This approach is often adopted for structure design because it is computationally efficient to explore numerous combinations of environmental conditions and utilizes existing monitoring data routinely collected at a national scale. Applying the industry standard method to predict the mean wave overtopping discharge allows us to use photos of past events collected from social media to: (1) assess the suitability of the generic/statistically based approaches to flood hazard forecasting (used by the EA and LAs) for current conditions; and (2) recommend improvements that could be made for current or future conditions.

## Data Availability

The datasets generated during the current study are available in the British Oceanographic Data Centre repository, https://www.bodc.ac.uk/data/published_data_library/catalogue/10.5285/acd939f0-38e5-57b0-e053-6c86abc0aa19/.
